# Enhanced cellulase production in *Trichoderma reesei* RUT C30 via constitution of minimal transcriptional activators

**DOI:** 10.1186/s12934-018-0926-7

**Published:** 2018-05-17

**Authors:** Jiajia Zhang, Guoxiu Zhang, Wei Wang, Wei Wang, Dongzhi Wei

**Affiliations:** 1 State Key Lab of Bioreactor Engineering, New World Institute of Biotechnology, East China University of Science and Technology, 130 Meilong Road, P.O.B. 311, Shanghai, 200237 China; 2 State Key Lab of Bioreactor Engineering, East China University of Science and Technology, Shanghai, 200237 China

**Keywords:** Minimal transcriptional activator, Cellulase production, *Trichoderma reesei*, ACEII, CREI

## Abstract

**Background:**

Cellulase can convert lignocellulosic feedstocks into fermentable sugars, which can be used for the industrial production of biofuels and chemicals. The high cost of cellulase production remains a challenge for lignocellulose breakdown. *Trichoderma reesei* RUT C30 serves as a well-known industrial workhorse for cellulase production. Therefore, the enhancement of cellulase production by *T. reesei* RUT C30 is of great importance.

**Results:**

Two sets of novel minimal transcriptional activators (DBD_ace2_-VP16 and DBD_cre1_-VP16) were designed and expressed in *T. reesei* RUT C30. Expression of DBD_ace2_-VP16 and DBD_cre1_-VP16 improved cellulase production under induction (avicel or lactose) and repression (glucose) conditions, respectively. The strain T_MTA66_ under avicel and T_MTA139_ under glucose with the highest cellulase activities outperformed other transformants and the parental strain under the corresponding conditions. For T_MTA66_ strains, the highest FPase activity was approximately 1.3-fold greater than that of the parental strain RUT C30 at 120 h of cultivation in a shake flask using avicel as the sole carbon source. The FPase activity (U/mg biomass) in T_MTA139_ strains was approximately 26.5-fold higher than that of the parental strain RUT C30 at 72 h of cultivation in a shake flask using glucose as the sole carbon source. Furthermore, the crude enzymes produced in the 7-L fermenter from T_MTA66_ and T_MTA139_ supplemented with commercial β-glucosidase hydrolyzed pretreated corn stover effectively.

**Conclusions:**

These results show that replacing natural transcription factors with minimal transcriptional activators is a powerful strategy to enhance cellulase production in *T. reesei*. Our current study also offers an alternative genetic engineering strategy for the enhanced production of industrial products by other fungi.

**Electronic supplementary material:**

The online version of this article (10.1186/s12934-018-0926-7) contains supplementary material, which is available to authorized users.

## Background

Lignocellulosic feedstocks are abundant and renewable resources in nature. They have been used to produce environment-friendly biofuels and chemicals, which have received increased attention for research [1]. The conversion of lignocellulosic feedstocks into fermentable sugars involving cellulase-based enzymatic saccharification is the key issue in large-scale production of biofuels and chemicals [2]. However, the industrial-scale production of biofuels and chemicals is limited in part by the high cost of cellulase enzymes [3–5]. *Trichoderma reesei*, which produces the enzymes necessary for the complete hydrolysis of lignocellulose, has been widely used for the production of commercial cellulase [6]. *T. reesei* RUT C30 has been proven to be a cellulase hyper-producer [7]. Therefore, improvement of cellulase production in *T. reesei* RUT C30 as a cellulosic biorefinery is of great importance.

The cellulase produced in *T. reesei* RUT C30 mainly comprise two cellobiohydrolases (CBHI and CBHII), two endoglucanases (EGI and EGII), and β-glucosidase I (BGLI) that synergistically hydrolyze lignocellulosic materials, together with related xylanases and auxiliary proteins [7–9]. Among the total proteins produced by *T. reesei*, CBHI and CBHII account for approximately 50–60 and 10–15%, respectively [10]. EGI and EGII are the two main endoglucanases and together account for 6–20% of the total produced proteins [11]. BGLI is the key enzyme involved in the complete conversion of cellobiose to glucose [12]. XYNI and XYNII are the main components of xylanase from *T. reesei* [13]. Additionally, swollen in, as a non-enzymatic cellulose attacking enzyme, and lytic polysaccharide monooxygenases (LPMOs) synergistically degrade lignocellulosic materials as auxiliary proteins [14, 15].

Expression of cellulase and xylanase in *T. reesei* is regulated by a combination of transcription factors such as XYRI, ACEII, and CREI. Among these factors, XYRI (xylanase regulator 1) is recognized as the primary transcriptional activator with a GAL4-like Zn(II)_2_Cys_6_ binuclear cluster domain [16]. Similar to XYRI, ACEII also acts as a transcriptional activator, consisting of a Zn(II)_2_Cys_6_ DNA-binding domain, which is responsible for the regulation of cellulase-encoding genes in *T. reesei* under induction conditions [17]. On the other hand, CREI is the primary negative regulator that mediates carbon catabolite repression (CCR). The expression of cellulase in *T. reesei* RUT C30 is inhibited by CREI when glucose is present [18].

In addition to protein engineering of cellulase chimeras to enhance specific activity [19], increasing cellulase production via the application of artificial transcription factors is an effective strategy [20–22]. An artificial transcription activator containing the two DNA-binding domains from CREI and ACEI and an effector domain from ACEII can regulate gene expression in *T. reesei* [20]. Zhang et al. [21] screened a mutant *T. reesei* strain U3 with enhanced cellulase production by transforming an artificial zinc finger protein library. Similarly, an artificial transcription activator linking the CREI-binding domain with the C-terminus of XYRI was shown to improve cellulase production in the recombinant strain *T. reesei* zxy-2 with glucose as the sole carbon source [22].

Here, we developed two sets of novel artificial transcriptional activators, which were designed as minimal transcriptional activators containing one DNA binding domain from either ACEII or CREI, and one strong transcriptional activation domain VP16 from the herpes simplex virus [23]. These minimal transcriptional activators DBD_ace2_-VP16 and DBD_cre1_-VP16 were transformed into *T. reesei* RUT C30 to replace the native regulators ACEII and CREI, respectively, in the genome by homologous double exchange. We then investigated the effects of these minimal transcriptional activators (DBD_ace2_-VP16 under induction conditions and DBD_cre1_-VP16 under repression conditions) on cellulase and xylanase generation. We suggest that the minimal transcriptional activators DBD_ace2_-VP16 and DBD_cre1_-VP16 can enhance cellulase production.

## Results

### Construction of transformants with minimal transcriptional activators

**Fig. 1:**
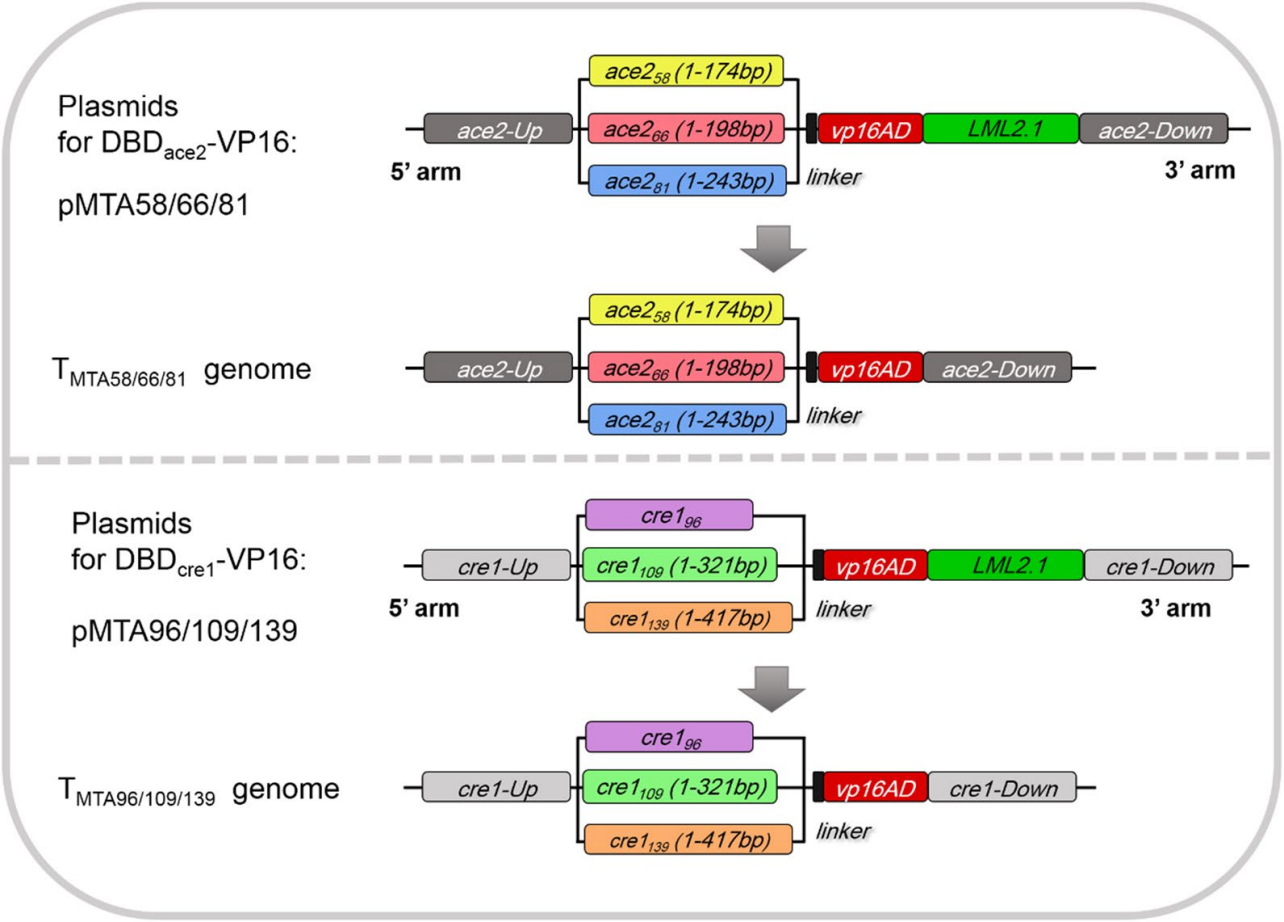
Construction of the minimal transcriptional activators. Plasmids for two sets of novel minimal transcriptional activators fusing the DNA-binding domains of ACEII or CREI with the VP16 activation domain and a short flexible linker (GGGGSGGGGS). Transformants T_MTA58/66/81_ and T_MTA96/109/139_ were obtained after xylose-induced marker rescue. The natural factors were replaced by the minimal transcriptional activators in transformants genomes

Each compact minimal transcription activator consists of one DNA binding domain (DBD) of the native transcription factor and the transcriptional activation domain VP16. The transcription factors ACEII and CREI were used as targets to be replaced. Three sets of *ace2* DBDs with different lengths were selected to construct the DBD_ace2_-VP16 (MTA58/66/81) minimal transcriptional activators. DBD_*ace2*–*58*_ (amino acids 1–58) is the core DBD as previously demonstrated using DNA mobility shift assays [17]. DBD_*ace2*–*66*_ (amino acids 1–66) includes a histidine-rich region (amino acids 53–66) with unknown function that is also found in other Zn(II)_2_Cys_6_-type regulatory proteins [17]. DBD_*ace2*–*81*_ (amino acids 1–81) is the longest region responsible for DNA binding and is followed by the region corresponding to the activation domain. Similarly, three sets of *cre1* DBDs were selected (*cre1*_*96*_, DBD_*cre1*–*109*_, and DBD_*cre1*–*139*_). However, *cre1*_*96*_ does not represent the DNA sequence of amino acids 1–96 of CREI but is a chimera due to the loss of a 2477-bp fragment at position + 262 of *cre1* in the *T. reesei* hyper-cellulolytic mutant RUT C30 [24]. The plasmids for minimal transcriptional activators DBD_ace2_-VP16 (pMTA58/66/81) and DBD_cre1_-VP16 (pMTA96/109/139) (Fig. 1) were transformed into *T. reesei* RUT C30 to replace the original transcriptional factors (*ace2* and *cre1*_*96*_, respectively) by homologous double exchange and eliminate the risk of any unpredictable mutagenesis caused by random insertion. We screened nine transformants each for DBD_ace2_-VP16 (named T_MTA58_-1/-2/-3, T_MTA66_-1/-2/-3, and T_MTA81_-1/-2/-3) and for DBD_cre1_-VP16 (named T_MTA96_-1/-2/-3, T_MTA109_-1/-2/-3, and T_MTA139_-1/-2/-3). All transformants were identified as correct gene knockout strains harboring single-copy DNA integration (Additional file 1: Figure S1). The plasmids and transformants are listed in Table 1.

**Table 1:** Plasmids and transformants for minimal transcriptional activators in this study

Minimal transcriptional activators	Plasmids	Transformants
DBD_ace2_-VP16	pMTA58	T_MTA58_-1/-2/-3
	pMTA66	T_MTA66_-1/-2/-3
	pMTA81	T_MTA81_-1/-2/-3
DBD_cre1_-VP16	pMTA96	T_MTA96_-1/-2/-3
	pMTA109	T_MTA109_-1/-2/-3
	pMTA139	T_MTA139_-1/-2/-3

### Growth of minimal transcriptional activator transformants

**Fig. 2:**
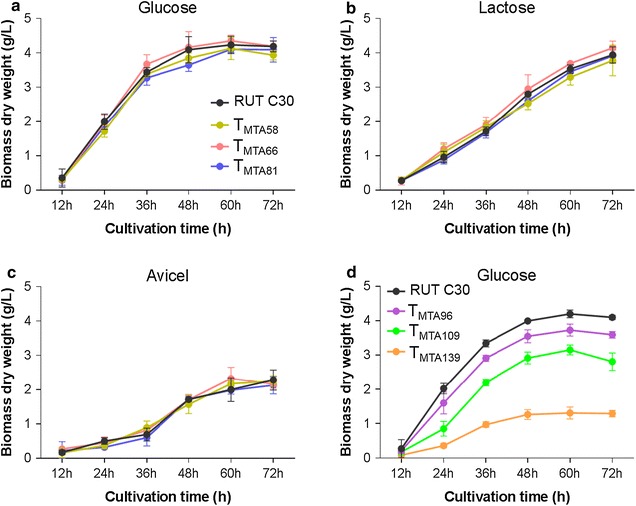
Cell growth differences between transformants and RUT C30. Conidia (10^6^/mL) of strains were incubated on MM medium supplemented with different carbon sources (2% w/v) for 72 h. RUT C30 and DBD_ace2_-VP16 transformants were cultured on glucose (**a**), lactose (**b**), and avicel (**c**). RUT C30 and DBD_cre1_-VP16 transformants were cultured on glucose (**d**). The biomass dry weight per liter was calculated from the intracellular protein content for avicel based on 0.32 g of intracellular protein per g dry biomass. Error bars show the respective standard deviation of three biological replicates

To determine whether the minimal transcriptional activators were involved in cellulase production under induction or repression conditions, we first investigated the growth of the transformants of the minimal transcriptional activators, along with the parental strain RUT C30, in glucose, lactose, and avicel. The growth of DBD_ace2_-VP16 transformants T_MTA58_, T_MTA66_, and T_MTA81_ were examined in minimal medium (MM) with glucose, lactose, or avicel as the sole carbon source (Fig. 2a–c). The growth of DBD_cre1_-VP16 transformants T_MTA96_, T_MTA109_, and T_MTA139_ were detected in MM containing glucose as the sole carbon source (Fig. 2d). The growth of DBD_ace2_-VP16 transformants exhibited no significant differences from the parental strain RUT C30 when cultured in glucose (Fig. 2a), lactose (Fig. 2b), or avicel (Fig. 2c). This suggests that DBD_ace2_-VP16 is not involved in basic cellular metabolism. However, the growth of DBD_cre1_-VP16 transformants T_MTA96_, T_MTA109_, and T_MTA139_ showed delayed growth in glucose (Fig. 2d). Growth delays were more pronounced in T_MTA139_ strains than those in the T_MTA96_ and T_MTA109_ strains, indicating that DBD_cre1_-VP16 affected the primary metabolism of the cells in glucose.

### Minimal transcriptional activator DBD_ace2_-VP16 promotes cellulase production under induction conditions

**Fig. 3:**
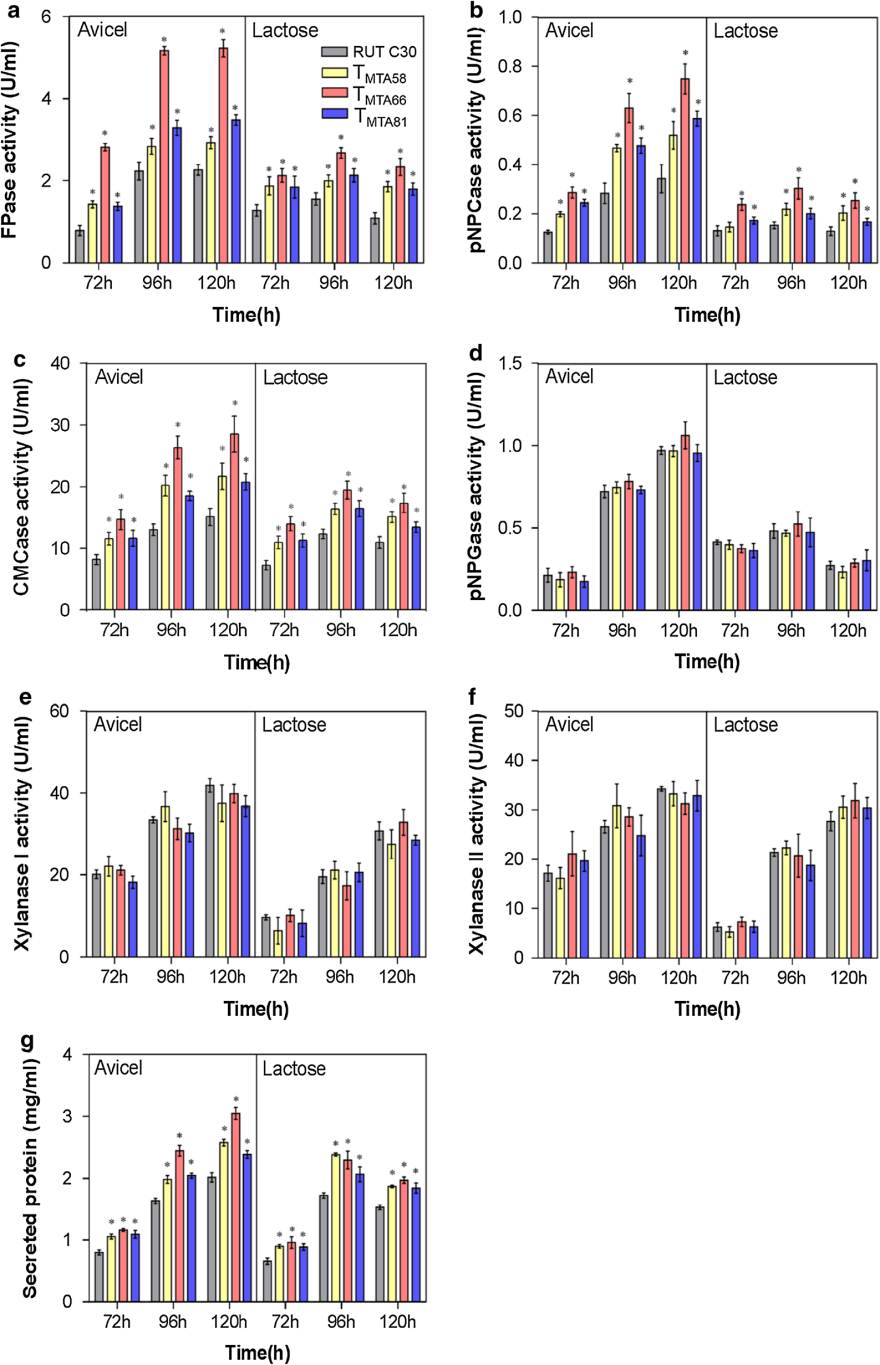
Cellulase and xylanase production in DBD_ace2_-VP16 transformants. DBD_ace2_-VP16 transformants and RUT C30 were cultured on 2% avicel or lactose after a shift from glucose. FPase (**a**), *p*NPCase (**b**), CMCase (**c**), *p*NPGase (**d**), xylanase I (**e**), and xylanase II (**f**) activity (U/mL), and extracellular secreted protein concentration (**g**) were measured at 72, 96, and 120 h. Error bars show the respective standard deviation of three biological replicates; asterisks indicate significant differences (*p ≤ 0.05) between the transformants and the parental strain RUT C30, as assessed by Student’s *t* test

To identify whether the minimal transcriptional activator DBD_ace2_-VP16 can enhance cellulase production, cellulase activities, xylanase activities, and secreted protein concentrations from transformants and parental RUT C30 were examined in induction conditions (avicel and lactose) (Fig. 3). Distinctly, the *p*NPCase, FPase, and CMCase activities of T_MTA58_, T_MTA66_, and T_MTA81_ in both lactose- and avicel-containing media were superior compared to those of the parental strain RUT C30 (Fig. 3a–c). Moreover, the tested transformants exhibited better performance in avicel than in lactose. The FPase activity in T_MTA66_ strains cultured in avicel at 120 h was 5.2 U/mL, which was about 1.3-fold greater than that produced by RUT C30 (Fig. 3a). In addition, T_MTA66_ strains also showed the highest *p*NPCase (0.7 U/mL) and CMCase activities (28.6 U/mL), which were about 2.2- and 1.9-fold, respectively, when compared to those of RUT C30 in avicel at 120 h (Fig. 3b, c). On the other hand, the *p*NPGase and xylanase activities had no significant difference between T_MTA58_, T_MTA66_, T_MTA81_, and RUT C30 (Fig. 3d–f). In agreement with the noticeable increment of cellulase activities, 51% more secreted protein was detected in the culture supernatant of T_MTA66_ transformants compared to that of the parental strain RUT C30 (Fig. 3g).

**Fig. 4:**
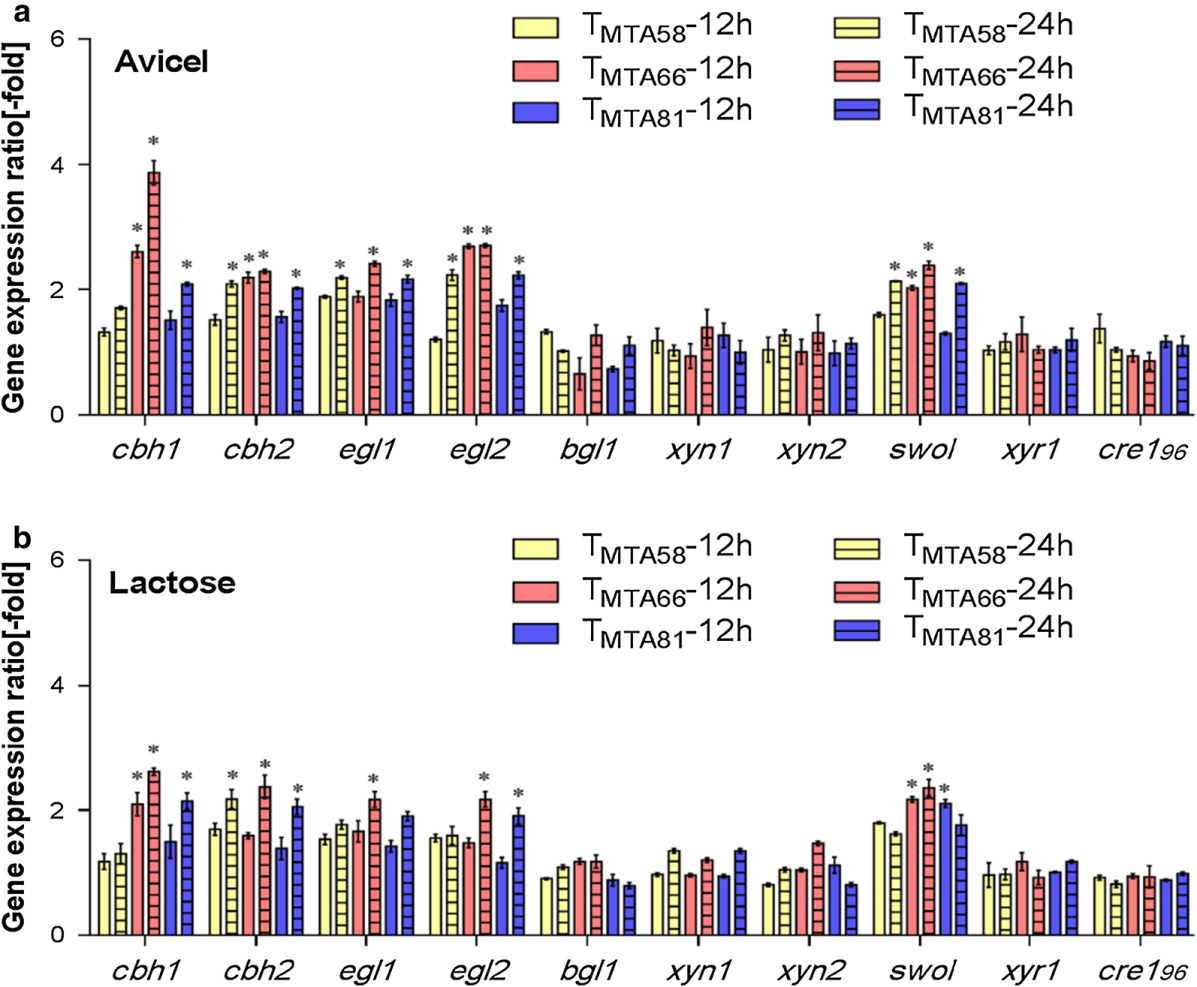
Comparison of transcript levels of main cellulase, xylanase, and transcription factors genes in DBD_ace2_-VP16 transformants. Gene expression ratios (-fold) in DBD_ace2_-VP16 transformants relative to RUT C30 on 2% avicel (**a**) and lactose (**b**) for 12 and 24 h after a shift from glucose. Gene expression ratios (-fold) were normalized to the corresponding gene expression at the same timepoint in the parental strain RUT C30. Values represent the mean of three biological replicates and error bars show the standard deviation; asterisks indicate significant differences (gene expression ratio greater than twofold or less than 0.5-fold between the transformants and the parental strain RUT C30)

To further confirm the effects of DBD_ace2_-VP16 on the synthesis of cellulase or total protein secretion, the transcript levels of cellulase-related genes including five main cellulase genes (*cbh1*, *cbh2*, *egl1*, *egl2*, and *bgl1*), two major xylanase genes (*xyn1* and *xyn2*), one accessary protein gene (*swo1*), and two transcription factor genes (*xyr1* and *cre1*_*96*_) at 12 and 24 h were analyzed using RT-qPCR (Fig. 4). T_MTA58_, T_MTA66_, and T_MTA81_ exhibited increased expression of cellulase genes *cbh1*, *cbh2*, *egl1*, and *egl2* compared to that of the parental strain in both lactose- and avicel-containing media (Fig. 4a, b), which is consistent with the result of enhanced cellulase activities. Moreover, avicel induced a higher ratio for cellulase gene expression than lactose. Notably, T_MTA66_ strains showed the strongest transcriptional activation among the strains. Using avicel as the sole carbon source, the transcript levels of *cbh1*, *cbh2*, *egl1*, and *egl2* in the T_MTA66_ strain increased about 2.9-, 1.3-, 1.4-, and 1.7-fold, respectively, when compared to that in *T. reesei* RUT C30 at 24 h (Fig. 4a). While, the transcript levels of *bgl1*, *xyn1*, and *xyn2* were not significantly upregulated in both avicel and lactose (Fig. 4a, b). We also detected an enhancement of transcript levels of *swo1* compared to that in parental strain (Fig. 4a, 4b). In addition, the transcript levels of *xyr1* and *cre1*_*96*_ showed no significant differences in the T_MTA58_, T_MTA66_, and T_MTA81_ transformants in comparison to that in RUT C30. This indicates that the minimal transcriptional activator DBD_ace2_-VP16 contributes to the expression of the cellulase genes due to the enhancement of its own transcriptional activation.

### Minimal transcriptional activator DBD_cre1_-VP16 promotes the cellulase and xylanase production under repression conditions

**Fig. 5:**
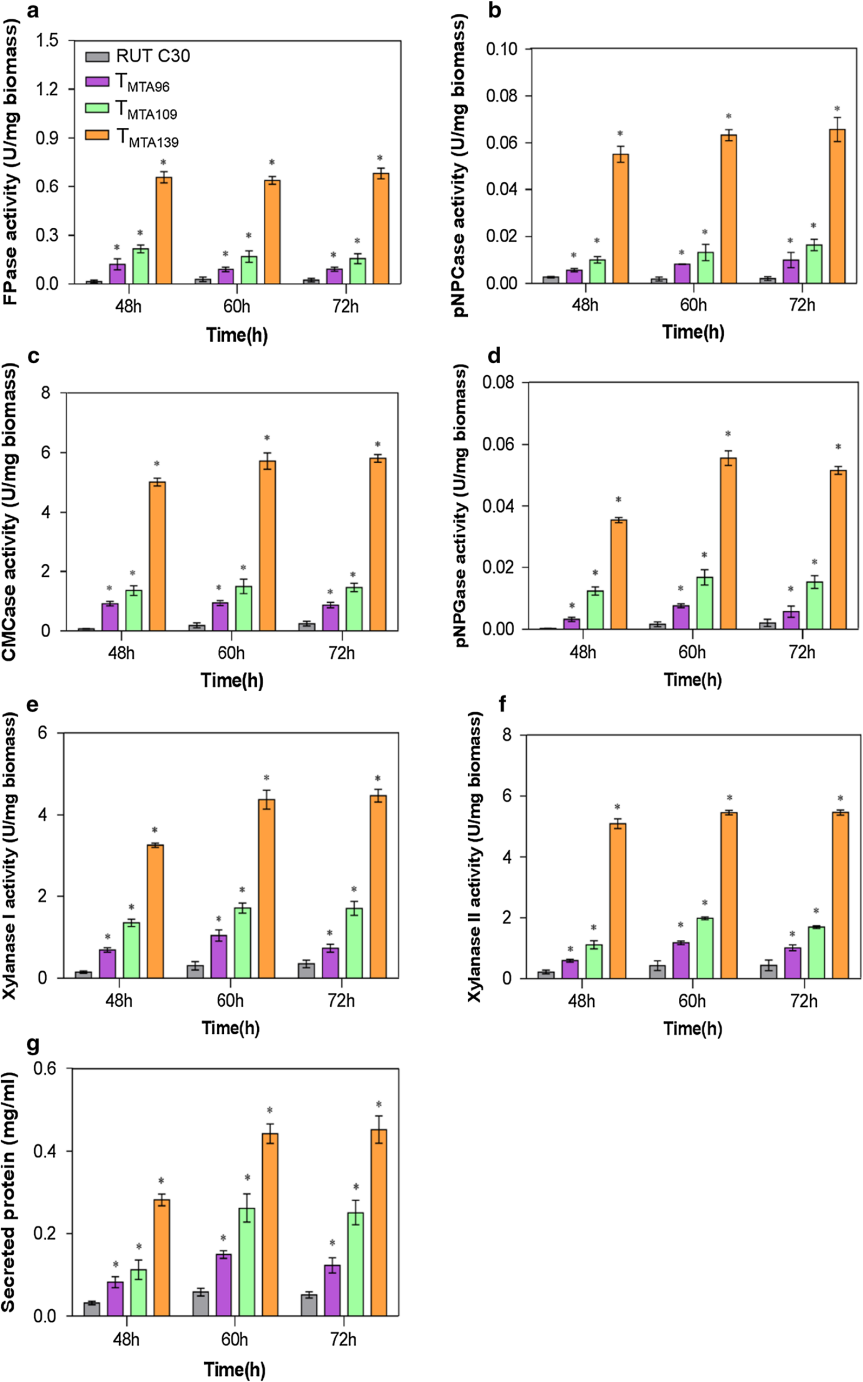
Cellulase and xylanase production in DBD_cre1_-VP16 transformants. DBD_cre1_-VP16 transformants and RUT C30 were cultured on 2% glucose. FPase (**a**), *p*NPCase (**b**), CMCase (**c**), *p*NPGase (**d**), xylanase I (**e**), and xylanase II (**f**) activity (U/g biomass), and extracellular secreted protein concentration (**g**) were measured at 48, 60, and 72 h. Error bars show the respective standard deviation of three biological replicates; asterisks indicate significant differences (*p ≤ 0.05) between the transformants and the parental strain RUT C30, as assessed by Student’s *t* test

**Fig. 6:**
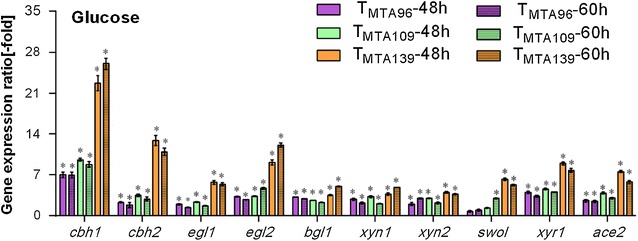
Comparison of transcript levels of main cellulase, xylanase, transcription factor genes in DBD_cre1_-VP16 transformants. Gene expression ratios (-fold) in DBD_cre1_-VP16 transformants relative to RUT C30 on 2% glucose for 48 and 60 h. Gene expression ratios (-fold) were normalized to the corresponding gene expression at the same timepoint in the parental strain RUT C30. Values represent the mean of three biological replicates and error bars show the standard deviation; asterisks indicate significant differences (gene expression ratio greater than twofold or less than 0.5-fold between the transformants and the parental strain RUT C30)

Given that CREI has been identified as a transcription factor responding to carbon source metabolite repression, glucose was used as the sole carbon source to analyze the effects of the minimal transcriptional activator DBD_cre1_-VP16 in the T_MTA96_, T_MTA109_, and T_MTA139_ transformants (Figs. 5, 6). Considering the growth delay of the T_MTA96_, T_MTA109_, and T_MTA139_ transformants compared to that of the parental strain RUT C30 in liquid culture (Fig. 2d), FPase, *p*NPCase, CMCase, and *p*NPGase activities were shown as U per mg *T. reesei* biomass to compare the differences in cellulase activities (Fig. 5). In T_MTA96_, T_MTA109_, and T_MTA139_, FPase, *p*NPCase, CMCase, and *p*NPGase activities were remarkably higher under glucose repression compared to those in the parental strain RUT C30 (Fig. 5a–d). The T_MTA139_ strain displayed the highest constitutive cellulase production using glucose as the sole carbon source compared to the other strains, with an FPase activity titer that was almost 26.5-fold higher than that obtained in *T. reesei* RUT C30 in shake flask culture at 72 h (Fig. 5a). The *p*NPCase (Fig. 5b), CMCase (Fig. 5c), and *p*NPGase activities (Fig. 5d) in T_MTA139_ were 0.07 U/mg biomass, 5.8 U/mg biomass and 0.05 U/mg biomass, which were about 31.5-, 22.4- and 24.4-fold, respectively, greater than that produced by *T. reesei* RUT C30 at 72 h. Moreover, the xylanase I (4.5 U/mg biomass) and xylanase II activities (5.5 U/mg biomass) in T_MTA139_ were approximately 11.8- and 11.5-fold, respectively, greater than that of RUT C30 at 72 h. Additionally, the extracellular protein concentration of T_MTA96_, T_MTA109_, and T_MTA139_ in glucose also increased 1.4-, 3.8-, and 7.7-fold compared to that of RUT C30 at 72 h (Fig. 5e), respectively, revealing that the protein production in T_MTA96_, T_MTA109_, and T_MTA139_ was improved in glucose. It could be confirmed by the SDS-PAGE (sodium dodecyl sulfate–polyacrylamide gel electrophoresis) assay (Additional file 1: Figure S2).

To further investigate the effects of DBD_cre1_-VP16 on the synthesis of cellulase and xylanase, transcript levels of target genes at 48 and 60 h were analyzed using RT-qPCR (Fig. 6). The transcript levels of *cbh1*, *cbh2*, *egl1*, *egl2*, and *bgl1* were markedly elevated in the T_MTA96_, T_MTA109_, and T_MTA139_ strains when cultivated in glucose, which is consistent with the observed increased cellulase activities. Among the DBD_cre1_-VP16 transformants, T_MTA139_ showed the best performance. The transcript levels of *cbh1*, *cbh2*, *egl1*, *egl2*, and *bgl1* in the T_MTA139_ strain were upregulated 25.1-, 9.9-, 4.4-, 11.1-, and 4.0-fold, respectively at 60 h compared to those in the parental strain RUT C30 (Fig. 6). Moreover, the transcript levels of *xyn1* and *xyn2* in the T_MTA139_ strain were significantly upregulated 3.9- and 2.7-fold compared to that in RUT C30 at 60 h (Fig. 6). On the other hand, there was a notable increase in the transcription of *xyr1* and *ace2* in the DBD_cre1_-VP16 transformants (Fig. 6). In the T_MTA139_ strain, the transcript levels of *xyr1* and *ace2* were 6.8- and 4.8-fold, respectively, greater than that in RUT C30 at 60 h. The enhanced transcription of *xyr1* and *ace2* may account for the elevated transcript levels of *cbh1*, *cbh2*, *egl1*, *egl2*, and *bgl1* [16, 17]. Additionally, the strong enhancement of the transcript levels of the swollenin gene *swo1* (Fig. 6) was detected in the T_MTA96_, T_MTA109_, and T_MTA139_ strains.

### Cellulase production in a jar fermentor and hydrolysis of corn stover by cellulase of T_MTA66_ and T_MTA139_ transformants

The strains T_MTA66_ in avicel and T_MTA139_ in glucose with the highest cellulase activities, consistent with the transcript levels, outperformed the other transformants and the parental strain RUT C30 in terms of cellulase production. Cellulase production by the T_MTA66_ and T_MTA139_ strains was further explored using a jar fermenter. The amount of secreted proteins and cellulase was significantly higher in these two strains than those in RUT C30 (Table 2). The T_MTA66_ strain showed maximum *p*NPCase (3.9 U/mL) and FPase (22.4 U/mL) activities after 5 days of cultivation. The T_MTA139_ strain reached an FPase activity of 4.5 U/mL after 3 days of cultivation (Table 2).

**Table 2:** Extracellular secreted protein and cellulase activities of the parental strain RUT C30, T_MTA66_, and T_MTA139_ in a 7-l jar fermenter after cultivation

Strain	Protein concentration (mg/mL)	Enzyme activity (U/mL)	
		FPase	*p*NPCase
RUT C30 (avicel)	6.2 ± 0.2	14.4 ± 0.2	2.3 ± 0.1
T_MTA66_ (avicel)	7.9 ± 0.2	22.4 ± 0.3	3.9 ± 0.1
RUT C30 (glucose)	1.4 ± 0.2	0.4 ± 0.2	0.1 ± 0.0
T_MTA139_ (glucose)	2.0 ± 0.1	4.5 ± 0.2	0.6 ± 0.0

**Fig. 7:**
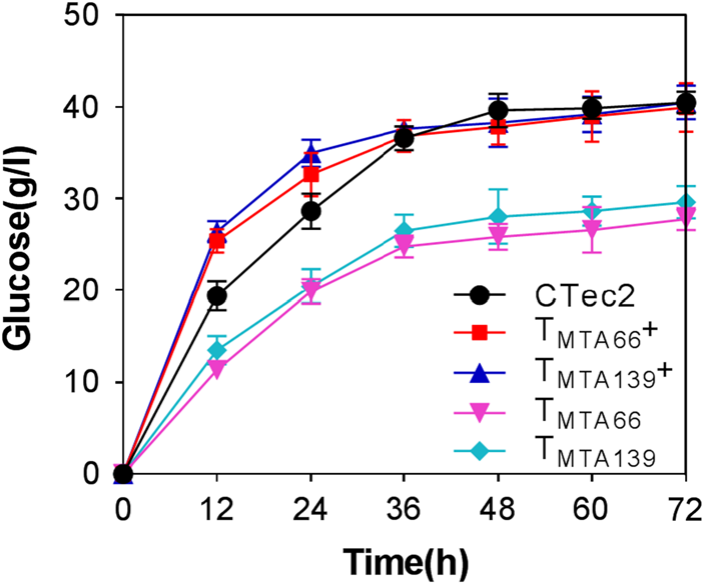
Hydrolysis of pretreated corn stover by CTec2 and the crude enzyme from T_MTA66_ and T_MTA139_ using the same FPase loading. The crude enzymes from T_MTA66_ and T_MTA139_ were either supplemented with β-glucosidase (SUNSON^®^) (T_MTA66_+, T_MTA139_+) or without β-glucosidase (T_MTA66_, T_MTA139_) for a CBU/FPA ratio of 2. The commercial cellulase CTec2 (Novozymes) was used as the control. Enzyme was supplemented at 15 FPA/g biomass. Values represent the mean and standard deviation of triplicate measurements

Saccharification by cellulase from T_MTA66_ and T_MTA139_ was determined via the hydrolysis of pretreated and biodetoxified corn stover [25]. The pretreated corn stover was first hydrolyzed to cellobiose during the catalysis of CBH and EG, and then cellobiose was hydrolyzed to glucose by ΒGL. Using the same FPase loading (15 U/g pretreated corn stover) without supplementation of commercial β-glucosidase, the glucose yields of the T_MTA66_ and T_MTA139_ strains showed similar low performances in saccharification of biomass to the glucose yield (66–71%) of the parental strain due to the lack of β-glucosidase produced by *T. reesei* (Fig. 7, Table 3, Additional file 1: Figure S3). With supplementation of commercial β-glucosidase, the glucose yields (98–99%) were almost the same as that obtained with the commercial enzyme CTec2 (Fig. 7, Table 3, Additional file 1: Figure S3). These results demonstrated that the enzymes from T_MTA66_ or T_MTA139_ supplemented with β-glucosidase were effective in hydrolyzing the pretreated corn stover.

**Table 3:** Saccharification of corn stover by the parental strain and transformants with equal FPA of 15 U/g biomass

Strain	FPA (U/g biomass)^a,b^	CBU (U/g biomass)^b^	Glucose (g/L)^a^	Glucose yield (%)^a^	Glucose yield (%)^b^
CTec2	15	30	40.5 ± 1.2	97 ± 2.9	ND
RUT C30 (avicel)	15	30	28.1 ± 1.3	67 ± 3.1	ND
T_MTA66_	15	30	27.8 ± 1.2	66 ± 2.9	98 ± 1.9%
T_MTA139_	15	30	29.6 ± 1.8	71 ± 4.3	99 ± 1.2%

Data are expressed as the mean of three independent experiments

*ND* not detected

^a^Without supplementation with commercial β-glucosidase (SUNSON^®^)

^b^Supplemented with β-glucosidase (SUNSON^®^) with a ratio of CBU/FPA at 2

## Discussion

Cellulase and xylanase genes are majorly regulated at the transcript level in fungi [26]. In *T. reesei*, transcriptional activators play a significant role in the regulation of the production of cellulase and xylanase. Two important domains of transcription factors, the DNA-binding domain and the transcriptional domain, are essential for their function. Most of the transcription factors in *T. reesei* still retain other fungi-specific domains [16, 17, 27], which might have an on–off function for cellulase and xylanase regulation. The minimal transcriptional activators DBD_ace2_-VP16 and DBD_cre1_-VP16 we constructed retained the core DNA binding domains of ACEII and CREI, respectively, did not contain redundant on–off regulation domains, and was fused with a strong transcriptional activation domain, VP16. VP16 was selected because of its compact structure (only 82 amino acids long) and stronger transcriptional activation than that of ACEII [28]. Retaining the core DNA binding domain of ACEII and CREI enabled our minimal transcriptional activators to competitively bind to promoters instead of the native transcription factors. The replacement of the VP16 strong transcriptional activation domain enhanced the transcription of downstream genes. Meanwhile, minimal transcriptional activators eliminated redundant on–off regulation domains to keep on an ON state. Thus, the minimal transcription activator DBD_ace2_-VP16 increased cellulase expression under induction conditions (avicel and lactose) and DBD_cre1_-VP16 increased cellulase and xylanase expression under repression conditions (glucose).

Additionally, the minimal transcriptional activator DBD_ace2_-VP16 enhanced cellulase production under induction conditions (Fig. 3). The transcript levels of related genes in DBD_ace2_-VP16 transformants showed consistent results (Fig. 4). A previous study reported that the absence of *ace2* decreased the transcript levels of the cellulase genes *cbh1*, *cbh2*, *egl1*, and *egl2*, and the total cellulase activity was reduced by 30–70% under cellulose induction conditions [17]. Therefore, the minimal transcription activator DBD_ace2_-VP16 enhanced transcriptional activation compared to the original structure of ACEII, consequently increasing cellulase production. Interestingly, DBD_ace2_-VP16 did not elevate the production of β-glucosidase and DBD_ace2_-VP16 was ineffective in improving xylanase production. Deleting *ace2* has no effect on *xyn1* expression, according to a previous study [17]. It is feasible that xylanase production was not improved in DBD_ace2_-VP16 transformants because of this. ACEII, however, can affect the expression of *xyn2* [17, 29]. Phosphorylation and dimerization are required for ACEII to bind the *xyn2* promoter [29]. The minimal transcriptional activator DBD_ace2_-VP16 possibly impacted the phosphorylation and dimerization of the ACEII domain, inhibiting the enhancement of *xyn2* expression in DBD_ace2_-VP16 transformants. Moreover, the minimal transcriptional activator DBD_ace2_-VP16 increased the transcript levels of *swol* gene, which contributed to the increased cellulase activity.

CREI acts as a carbon catabolite repressor of cellulase and xylanase [30]. However, minimal transcriptional activator DBD_cre1_-VP16 yielded a positive effect on cellulase and xylanase production (Figs. 5, 6). Mello-de-Sousa et al. [24] reported that the truncated CREI-96 protein of RUT C30 exhibits a positive regulation on the expression of target genes. Moreover, the CREI-96 protein can bind to the promoter of *cbh1* and *cbh2*, resulting in increased gene expression with a simultaneous opening of chromatin structure in *xyr1* [24]. XYRI is considered as the major transcriptional activator for cellulase and xylanase expression. The absence of *xyr1* downregulates the expression of cellulase and xylanase in *T. reesei* [31]. Additionally, CREI binds to the promoter of *xyn1* [32], and carbon catabolite repression (CCR) mediated by CREI can lead to the downregulated expression of *xyn2* [33]. These explain our results regarding minimal transcriptional activator DBD_cre1_-VP16 acting as a positive regulator with enhanced *xyr1* expression to improve cellulase and xylanase production in glucose (Fig. 6).

However, our minimal transcriptional activator transformants require further improvements. Though the high-yielding T_MTA139_ strain displayed constitutive cellulase production, with the production titer being 26.5-fold greater than that in *T. reesei* RUT C30 (Fig. 5a), this strain showed poor stability. After three generations, the T_MTA139_ strain began to retrogress, and the total FPase activity titer was substantially reduced; on the contrary, the growth rate significantly increased. Further optimization of T_MTA139_ strain stability is expected to be done in future studies.

Zhang et al. [22] reported the overexpression of an artificial transcription activator for constitutive cellulase production, which is the first report on cellulase production from glucose by *T. reesei* RUT C30 engineered with an artificial transcription factor. In related studies, artificial transcription activators were randomly inserted into the genome of *T. reesei* [20–22], which caused collateral mutations. Collateral mutations resulting in high cellulase production in *T. reesei* have been reported [34, 35]. Future studies will focus on overexpression, random insertion, and the combination of two sets of minimal transcriptional activators to further enhance cellulase production of superior cellulase-producing strains.

Transcription factors are switches that finely regulate gene expression and they enable organisms to better adapt to a particular environment. Transcription factors in *T. reesei* still retain their original evolutionary state [27]. To produce more cellulase and hemicellulase, minimal transcriptional activators offer an alternative genetic engineering strategy, and may enhance the production of industrial products in other fungi.

## Conclusions

Minimal transcriptional activators were first constructed and transformed in *T. reesei* by fusing a DNA-binding domain of a native regulator with the VP16 activation domain to enhance cellulase production. Crude enzymes for both strains supplemented with commercial β-glucosidase were used to hydrolyze pretreated corn stover, and 98–99% glucose yields were obtained. These results demonstrate that replacing natural transcription factors with minimal transcription activators is a powerful strategy for enhancing cellulase production in *T. reesei* RUT C30. In addition, our current study also offers an alternative genetic engineering strategy for the enhanced production of industrial products in other fungi.

## Methods

### Strains

*Trichoderma reesei* RUT C30 (ATCC 56765), a hyper-cellulolytic strain, was used as the parental strain for genetic transformation and chromosomal DNA preparation. *Escherichia coli* DH5α and *Agrobacterium tumefaciens* AGL1 were used for plasmid construction and *Agrobacterium*-mediated transformation, respectively.

### Construction of minimal transcriptional activators

The upstream and downstream sequences of *ace2* and *cre1*_*96*_ genes were amplified from *T. reesei* RUT C30 genomic DNA through PCR using the appropriate primers and used as the 5′ and 3′ homology arms of pMTA58/66/81 and pMTA96/109/139, respectively (Fig. 1). The different truncated versions of the DNA-binding domain sequence of *ace2* (DBD_*ace2*–*58*_, DBD_*ace2*–*66*_, DBD_*ace2*–*81*_) and *cre*_*96*_ [24] were amplified from *T. reesei* RUT C30 genomic DNA via PCR, while the DBD_*cre1*–*109*_ and DBD_*cre1*–*139*_ (different truncated versions of the DNA-binding domain sequence of *cre1*) were cloned from *T. reesei* Qm9414 genomic DNA using the appropriate primers because the *cre1* in RUT C30 was truncated and chimeric. All primers are listed in Additional file 1: Table S1. Linker and VP16 activation domain sequences were amplified from pG1V [28] through PCR. The resulting fragments were sequentially fused to linearized LML2.1 (digested by *Pac*I/XbaI or *Swa*I) [36], by which resistance hygromycin B marker gene could be lost through xylose-induced Cre recombinase. All the vectors pMTA58, pMTA66, pMTA81, pMTA96, pMTA109, and pMTA139 (Fig. 1) were generated by the Seamless Cloning Kit (TransGen Biotech, Beijing, China). All plasmids were confirmed via DNA sequencing.

### Transformation of *T. reesei* and characterization of the transformants

The generated plasmids were introduced into *T. reesei* RUT C30 via *Agrobacterium*-mediated transformation [37]. Clone verification and xylose-induced marker rescue [36] were performed to obtain the final transformants T_MTA58/66/81_ and T_MTA96/109/139_. The integration of the target gene-deleted constructs was analyzed using diagnostic PCR and sequencing. The single-copy DNA integration in transformed clones was verified by quantitative PCR (qPCR). The details are listed in Additional file 1: Table S1. For each minimal transcription activator, three final transformants were randomly selected and subcultured for subsequent enzyme production.

### Biomass concentration assay

Conidia (10^6^/mL) of final transformants and the parental strain RUT C30 were inoculated into 100 mL of minimal medium ((NH_4_)_2_SO_4_ 5 g/L; Urea 0.3 g/L; KH_2_PO_4_ 15 g/L; CaCl_2_ 0.6 g/L; MgSO_4_ 0.6 g/L; FeSO_4_·7H_2_O 5 mg/L; ZnSO_4_·7H_2_O 1.4 mg/L; CoCl_2_·6H_2_O 2 mg/L, pH 5.5) supplemented with 20 g/L glucose, lactose, or avicel in 500-mL Erlenmeyer flasks and incubated at 28 °C and 200 rpm for 72 h. Two milliliters of the culture liquid was collected every 12 h for biomass concentration assay. Biomass concentrations from glucose and lactose were measured gravimetrically according to the method of Corder et al. [38]. Biomass formation from avicel were indirectly determined by the amount of intracellular protein, as previously reported [39]. In brief, harvested mycelia were suspended in 1 mL 1 M NaOH in a reaction tube and the mixture was incubated for 2 h frequently being vortexed. The suspension was clarified via centrifugation at 14,000×*g* at 4 °C for 10 min. Total protein concentration of the suspension was determined by the Modified Lowry Protein Assay Kit (Sangon Biotech, Shanghai, China). The final protein content was corrected using a set of substrate controls where no inoculum was added to the avicel medium. The biomass dry weight was then calculated assuming an average content of 0.32 g intracellular protein per g of dry cell mass. Each experiment was performed in three biological replicates.

### Cellulase production in a flask

To identify the cellulase production of T_MTA58/66/81_, 10^6^/mL conidia of *T. reesei* strains were inoculated into 100 mL of Mandels-Andreotti medium [40] supplemented with 2% (w/v) glucose in a 500-mL shake flask incubated at 28 °C and 200 rpm for 36 h. Vegetative mycelia (0.4 g biomass) were collected by filtration, washed with distilled water, dried with sterile filter paper, and then subcultured into fresh 100 mL of Mandels-Andreotti medium supplemented with 2% (w/v) lactose or avicel in a 500-mL shake flask at 28 °C and 200 rpm. For T_MTA96/109/139_, 10^6^/mL conidia of *T. reesei* strains were inoculated into 100 mL of Mandels-Andreotti medium supplemented with 2% (w/v) glucose in a 500-mL shake flask at 28 °C and 200 rpm for 72 h without medium replacement. Each experiment was performed in three biological replicates.

### RNA extraction, reverse transcription, and real-time quantitative PCR (RT-qPCR) analysis

Total RNA was extracted using FastRNA Pro Red Kit (MP Biomedicals, Santa Ana, CA, USA) according to the manufacturer’s instructions. Synthesis of cDNA with 1000 ng total RNA was performed using TransScript All-in-One First-Strand cDNA Synthesis SuperMix for qPCR (TransGen, Beijing, China) following the manufacturer’s instructions. The transcript levels of target genes were assessed using real-time quantitative PCR (RT-qPCR) and normalized to that of the *sar1* gene [41] using the 2^−ΔΔCt^ method. The cycling conditions comprised 30 s initial denaturation and polymerase activation at 95 °C, followed by 40 cycles of 5 s at 95 °C and 60 s at 64 °C via an ABI StepOne Plus thermocycler (Applied Biosystems, Foster City, CA, USA). The primers are described in Additional file 1: Table S1. Threshold cycle (Ct) values and PCR efficiencies were used to calculate relative expression quantities by the ABI software. All samples were detected in three independent experiments with three replicates.

### Enzyme assays, secreted protein concentration and SDS-PAGE assays

The supernatants collected via centrifugation (10,000×*g* for 10 min at 4 °C) were used for enzyme and secreted protein concentration assays. The FPase and CMCase activities were measured via the DNS method using glucose as a standard. One unit represents the amount of enzyme that formed 1 µmol of reducing sugar per minute during the hydrolysis reaction. The *p*NPCase and *p*NPGase activities were measured against *p*-nitrophenol-d-cellbioside (*p*NPC) and *p*-nitrophenyl β-d-glucopyranoside (*p*NPG) (Sigma-Aldrich, St. Louis, USA), respectively. One unit of *p*NPCase and *p*NPGase activity was defined as 1 μmol of *p*-nitrophenol released per minute during the hydrolysis reaction. Xylanase I and II activities were determined by xylan degradation at pH values of 3.7 and 5.0 [29], respectively. One unit of xylanase activity is defined as releasing 1 μmol of xylose reducing sugar equivalents per minute under the defined assay conditions. Protein concentration was determined using the Modified Lowry Protein Assay Kit (Sangon Biotech, Shanghai, China). All experiments were performed in three biological replicates. SDS-PAGE electrophoresis was carried out with 12% polyacrylamide separating gel.

### Cellulase production in a jar

Selected dominant strains T_MTA139_ and T_MTA66_ were cultivated in a 7-L jar fermenter (BIOTECH-5BG-7000, Baoxing BIO-ENGINEERING EQUIPMENT, shanghai, China) with a final working volume of 3 L. The cultivations T_MTA66_ and parental strain RUT C30 were performed as follows. Conidia (10^6^/mL) of strains were inoculated into 200 mL of Mandels-Andreotti medium supplemented with 1% (w/v) glucose and 1% (w/v) avicel in a 1-L shake flask and subsequently incubated with shaking (200 rpm) at 28 °C for 2 days. The culture was added to 2.8-L of fresh Mandels-Andreotti medium supplemented with 6% (w/v) avicel in a jar fermenter. Cultivation was carried out at 28 °C with 25% dissolved oxygen and 2 vvm (volumes of air per volume of liquid per minute) of aeration for 5 days. The pH was controlled within the range of 4.0–4.3 for the first 2 days and 5.0–5.2 thereafter.

The cultures of T_MTA139_ and the parental strain RUT C30 were performed as follows. Conidia (10^6^/mL) of strains were inoculated into 200 mL of Mandels-Andreotti medium supplemented with 2% (w/v) glucose in a 1-L shake flask and incubated with shaking (200 rpm) at 28 °C for 2 days. The culture was poured into 2.8 L of Mandels-Andreotti medium fresh supplemented with 2% (w/v) glucose in the jar fermenter. Cultivation was carried out at 28 °C with 25% dissolved oxygen and 2 vvm of aeration for 3 days. The pH was controlled within the range of 5.0–5.2.

### Enzymatic hydrolysis of corn stover by crude enzyme

Pretreated and biodetoxified corn stover was donated by Professor Jie Bao [25]. The pretreated corn stover was determined to contain 37.6% of cellulose and 4.4% hemicellulose in the dry mass. The crude enzymes produced by the *T. reesei* strains were supplemented with/without β-glucosidase (SUNSON^®^) with a CBU/FPA ratio of two to hydrolyze the corn stover. Hydrolysis experiments were performed in a flask containing 10% (w/v) pretreated corn stover as the substrate and FPase loading (15 U/g dry biomass) at 50 °C and pH 5.0 for 72 h. The methods for glucose analysis and calculation of glucose yield were based on the study by Li et al. [42].

## Additional file

**Additional file 1: Table S1.** All primers used for this study. **Figure S1.** The verification of the single-copy DNA integration in transformed clones by diagnostic PCR and qPCR. **Figure S2.** SDS-PAGE analysis of extracellular proteins secreted by *T. reesei* RUT C30 and T_MTA96_, T_MTA109_, T_MTA139_. **Figure S3.** Hydrolysis of pretreated corn stover by CTec2 and the crude enzyme from T_MTA66_, T_MTA139_ and RUT C30 using the same FPase loading.

## Abbreviations

MTA: minimal transcriptional activators
CBH: cellobiohydrolases
EG: endoglucanases
BGL1: β-glucosidase I
FPA/FPase: the filter paper activity
CMCase: the CMC activity
*p*NPCase: the CBH activity
*p*NPGase: the β-glucosidase activity
CBU: cellobiase units
U: international units
DNS: 3,5-dinitrosalicylic acid
CCR: carbon catabolite repression
vvm: volumes of air per volume of liquid per minute
SDS-PAGE: sodium dodecyl sulfate–polyacrylamide gel electrophoresis
*p*NPC: *p*-nitrophenol-d-cellobioside
*p*NPG: 4-nitrophenyl-beta-d-galactopyranoside
CMC: sodium salt of caboxy methyl cellulose
LPMOs: lytic polysaccharide monooxygenases
