# Supplementary material for: Enhanced cellulase production in Trichoderma reesei RUT C30 via constitution of minimal transcriptional activators
Source: Microb Cell Fact. 2018 May 17;17:75. doi: 10.1186/s12934-018-0926-7 (PMC5956553; doi:10.1186/s12934-018-0926-7)
Supplement: Supplementary file 1 — Additional file 1: Table S1. All primers used for this study. Figure S1. The verification of the single-copy DNA integration in transformed clones by diagnostic PCR and qPCR. Figure S2. SDS-PAGE analysis of extracellular proteins secreted by T. reesei RUT C30 and TMTA96, TMTA109, TMTA139. Figure S3. Hydrolysis of pretreated corn stover by CTec2 and the crude enzyme from TMTA66, TMTA139 and RUT C30 using the same FPase loading. [file 12934_2018_926_MOESM1_ESM.docx]

**Additional file 1**

**Enhanced cellulase production** **in** ***Trichoderma reesei* RUT C30 via constitution of** **minimal** **transcriptional activators**

Jiajia Zhang^1^, Wei Wang^2^, Wei Wang^1^*, Dongzhi Wei^1^

1. State key Lab of Bioreactor Engineering, New World Institute of Biotechnology, East China University of Science and Technology, Shanghai 200237, China.

2. School of Pharmacy, East China University of Science and Technology, Shanghai 200237, China.

* Corresponding author: Wei Wang

Mailing address: East China University of Science and Technology, P.O.B. 311, 130 Meilong Road, Shanghai 200237, China

Phone: +86-21-64253287, Fax: +86-21-64250068.

E-mail: wadexp@ecust.edu.cn

**Table S1. All primers used for this study.**

| Primer | oligos Sequences (5’ to 3’) |
| --- | --- |
| **Quantitative RT-PCR analysis** | |
| Qsar1-1 | TGGATCGTCAACTGGTTCTACGA |
| Qsar1-2 | GCATGTGTAGCAACGTGGTCTTT |
| Qcbh1-1 | CTCCATCTCCGAGGCTCTTACC |
| Qcbh1-2 | GCAAGTGCCGCCATATCTGTTAT |
| Qcbh2-1 | GCATATTACGCCTCTGAAGTTAGCA |
| Qcbh2-2 | GCATAGTTACCGCCATTCTTGTTG |
| Qegl1-1 | AACTACCGCTGGATGCACGA |
| Qegl1-2 | TAGTCGACGCCCTCGATGAA |
| Qegl2-1 | TGAACAAGTCCGTGGCTCCAT |
| Qegl2-2 | ACAATTCGTAGGTCCGCTCCAA |
| Qbgl1-1 | CAAGTGACTGGTGCCGAGGTA |
| Qbgl1-2 | CGTTGCTGTTCCGCTCTGAC |
| Qxyn1-1 | GGTTGGACGACTGGATCT |
| Qxyn1-2 | GGTTGTCCTCCATGATGTAG |
| Qxyn2-1 | CATCGTCGAGAACTTTGGCA |
| Qxyn2-2 | GCGTGCGGTAAATGTCGTAG |
| Qswol-1 | CCAAACTATACGAGTAGCC |
| Qswol-2 | GAGTGAATGTCTTGATGG |
| Qxyr1-1 | CTTCCTCCTCCTGCTCATCG |
| Qxyr1-2 | TCGTGTGCCCTAACAATGGTC |
| Qace2-1 | TCAACATCCTCCACCACCAGTC |
| Qace2-2 | TGTCGGCGTACTCTCTCAGC |
| Qcre196-1 | GTCACCGTCATCAAGCCCAA |
| Qcre196-2 | AGGGCACTTGTAGGGACGAG |
| **Construction of MTA96 vector** | |
| Cre1-D1 | CGAATTCTTAATTAAGTTGAAATGGAAGCCATTGGAGCA |
| 96-D2 | CCGCCaagcttGAAAAAAAAGCAGGTAATGGAGGT |
| 96-D3 | TTTTCaagcttGGCGGTGGCGGAA |
| VP16-R | ACGAATAATTTCTAGATTAGCCGCCGTACTCGTCGATG |
| Cre1-R-1 | ACTAGTGAGCTCATTTCTGCCAATGTAGGTAAGTAGTAAGG |
| Cre1-R-2 | AGTGCCAAGCTTATTTGACGAGGACAAGGAAGGAGTT |
| **Construction of MTA109 vector** | |
| Cre1-D1 | CGAATTCTTAATTAAGTTGAAATGGAAGCCATTGGAGCA |
| 109-D2 | CCGCCaagcttGTGTATCCTCGAGTGCCTCGT |
| 109-D3 | ATACACaagcttGGCGGTGGCGGAA |
| VP16-R | ACGAATAATTTCTAGATTAGCCGCCGTACTCGTCGATG |
| Cre1-R-1 | ACTAGTGAGCTCATTTCTGCCAATGTAGGTAAGTAGTAAGG |
| Cre1-R-2 | AGTGCCAAGCTTATTTGACGAGGACAAGGAAGGAGTT |
| **Construction of MTA139 vector** | |
| Cre1-D1 | CGAATTCTTAATTAAGTTGAAATGGAAGCCATTGGAGCA |
| 139-D2 | CGCCaagcttGCCATCGACGTGCATGGGGT |
| 139-D3 | TCGATGGCaagcttGGCGGTGGCGGAA |
| VP16-R | ACGAATAATTTCTAGATTAGCCGCCGTACTCGTCGATG |
| Cre1-R-1 | ACTAGTGAGCTCATTTCTGCCAATGTAGGTAAGTAGTAAGG |
| Cre1-R-2 | AGTGCCAAGCTTATTTGACGAGGACAAGGAAGGAGTT |
| **Construction of MTA58 vector** | |
| Ace2-D1 | ACGAATTCTTAATTAATCCTGCTTCCTTCGGACTGT |
| 58-D2 | ACTGTGTTCATGGCTGTGGTT |
| 58-D3 | ACAGCCATGAACACAGTaagcttGGCGGTGGCGGAA |
| VP16-D4 | TGCTATACGAATAATTTCTAGATTAGCCGCCGTACTCGTCGATG |
| Ace2-R-1 | ACTAGTGAGCTCATTTTATGGACGGCAACGGTGAGG |
| Ace2-R-2 | AGTGCCAAGCTTATTTGGAGAAGCAACACGCATCAATCT |
| **Construction of MTA66 vector** | |
| Ace2-D1 | ACGAATTCTTAATTAATCCTGCTTCCTTCGGACTGT |
| 66-D2 | GTTATGATTGTGACTGTGACTATGACT |
| 66-D3 | ACAGTCACAATCATAACaagcttGGCGGTGGCGGAA |
| VP16-D4 | TGCTATACGAATAATTTCTAGATTAGCCGCCGTACTCGTCGATG |
| Ace2-R-1 | ACTAGTGAGCTCATTTTATGGACGGCAACGGTGAGG |
| Ace2-R-2 | AGTGCCAAGCTTATTTGGAGAAGCAACACGCATCAATCT |
| **Construction of MTA81 vector** | |
| Ace2-D1 | ACGAATTCTTAATTAATCCTGCTTCCTTCGGACTGT |
| 81-D2 | CTCCAAGCTCATGAGATCGAG |
| 81-D3 | ATCTCATGAGCTTGGAGaagcttGGCGGTGGCGGAA |
| VP16-D4 | TGCTATACGAATAATTTCTAGATTAGCCGCCGTACTCGTCGATG |
| Ace2-R-1 | ACTAGTGAGCTCATTTTATGGACGGCAACGGTGAGG |
| Ace2-R-2 | AGTGCCAAGCTTATTTGGAGAAGCAACACGCATCAATCT |

**Figure S1. The verification of the single-copy DNA integration in transformed clones by diagnostic PCR and qPCR.**

**a.** Schematic for identification of gene integration and single-copy DNA integration in transformants genome. Primers showed in green are used to verify the gene integration. Primers showed in red are used to identify the copy number of integrated genes. The verification of copy numbers for DBD_ace2_-VP16 (**b**) and DBD_cre1_-VP16 (**c**) in transformants by qPCR.

**Figure S2. SDS-PAGE analysis of extracellular proteins secreted by *T. reesei* RUT C30 and T_MTA96_, T_MTA109_, T_MTA139_.**

Equal volume of culture supernatant from *T. reesei* RUT C30, T_MTA96_, T_MTA109_, and T_MTA139_ cultured for 72 h in glucose was loaded in each lane. Molecular weight markers (lane M), cellulase and xylanase I and II are indicated.

**Figure S3**. **Hydrolysis of pretreated corn stover by CTec2 and the crude enzyme from T_MTA66_, T_MTA139_ and RUT C30 using the same FPase loading.**

The crude enzymes from T_MTA66_, T_MTA139_ and RUT C30 were either supplemented with β-glucosidase (SUNSON^®^) (T_MTA66_^+^, T_MTA139_^+^, RUT C30^+^) or without β-glucosidase (T_MTA66_, T_MTA139_, RUT C30) for a CBU/FPA ratio of 2. The commercial cellulase CTec2 (Novozymes) was used as the control. Enzyme was supplemented at 15 FPA/g biomass. Values represent the mean and standard deviation of triplicate measurements.

**Figure S1**


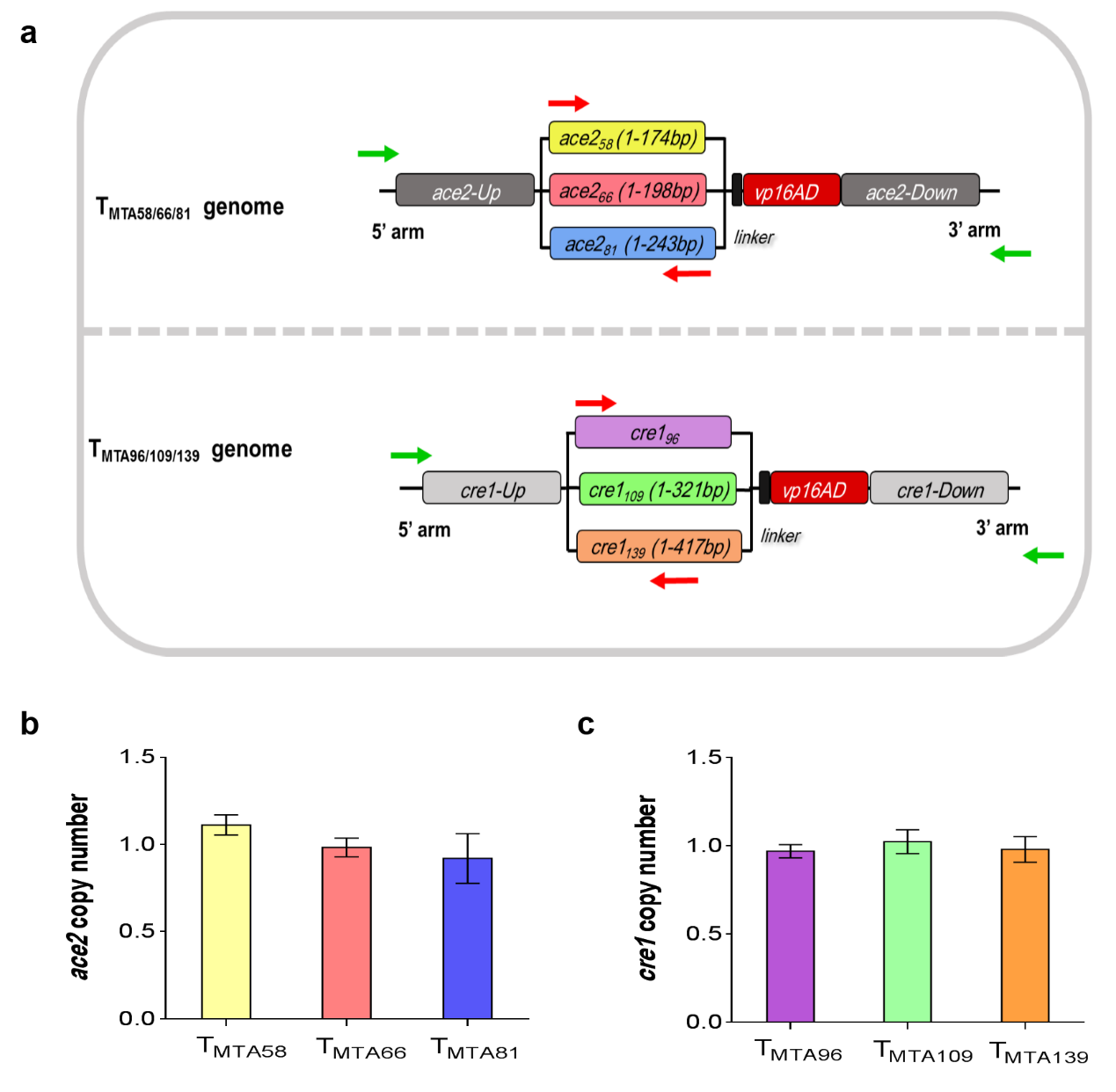


**Figure S2**

**
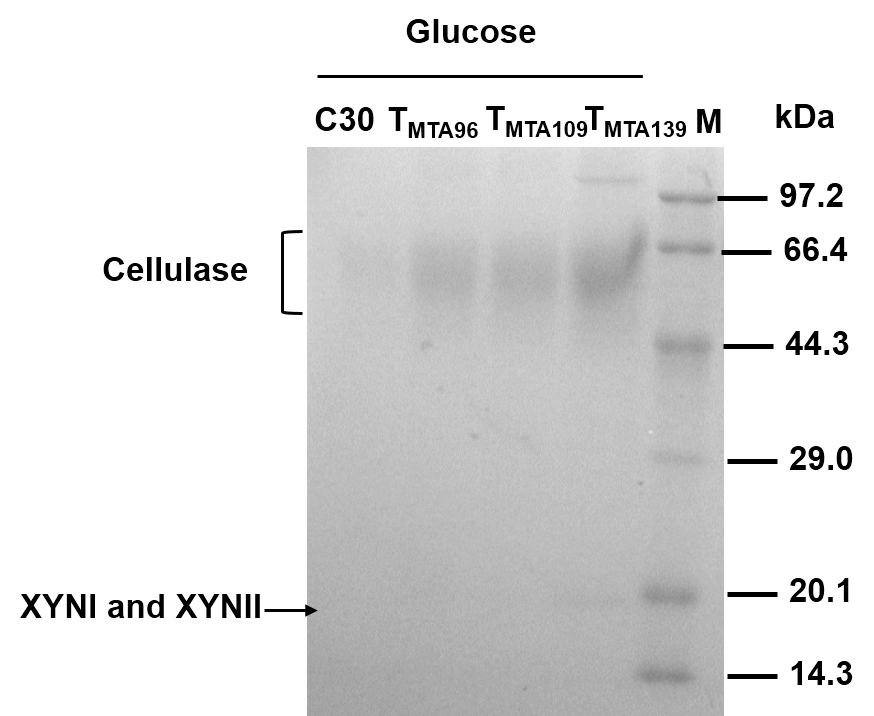
**

**Figure S3**

**
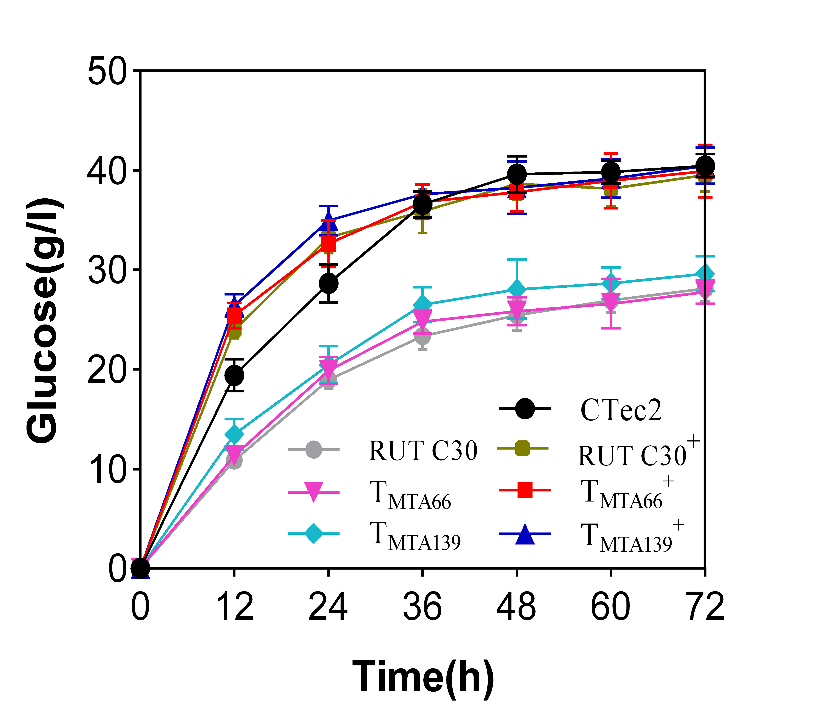
**
